# Evidence for a Numerosity Category that is Based on Abstract Qualities of “Few” vs. “Many” in the Bottlenose Dolphin (*Tursiops truncatus*)

**DOI:** 10.3389/fpsyg.2012.00473

**Published:** 2012-11-07

**Authors:** Sevgi Yaman, Annette Kilian, Lorenzo von Fersen, Onur Güntürkün

**Affiliations:** ^1^Marineland MallorcaMallorca, Spain; ^2^Abteilung Biopsychologie, Institut für Kognitive Neurowissenschaft, Fakultät für Psychologie, Ruhr-Universität BochumBochum, Germany; ^3^Tiergarten NürnbergNürnberg, Germany

**Keywords:** cetacea, numerosity, reversal learning, categorization, Weber-Fechner law

## Abstract

A previous study (Kilian et al., 2003) had demonstrated that bottlenose dolphins can discriminate visual stimuli differing in numerosity. The aim of the present study was twofold: first, we sought to determine if dolphins are able to use a numerical category based on “few” vs. “many” when discriminating stimuli according to the number of their constituent patterns. Second, we aimed to extend the previously demonstrated range of numbers, thereby testing the limits of the numerical abilities of bottlenose dolphins. To this end, one adult bottlenose dolphin learned to discriminate between two simultaneously presented stimuli which varied in the number of elements they contained. After initial training, several confounding parameters were excluded to render it likely that discrimination performance indeed depended on numerosity. Subsequently, the animal was tested with new stimuli of intermediate as well as higher numbers of elements. Once discrimination had been achieved, a reversal-training on a subset of stimuli was initiated. Afterward, the subject generalized the reversal successful to new and unreinforced stimuli. Our results reveal two main findings: firstly, our data strongly suggest a magnitude and a distance effect. Thus, coding of numerical information in dolphins might follow logarithmic scaling as postulated by the Weber-Fechner law. Secondly, after learning a reversal of contingencies, the dolphin generalized the reversal successful to new and unreinforced stimuli. Thus, within the limits of a study that was conducted with a single individual, our results suggest that dolphins are able to learn and use a numerical category that is based on abstract qualities of “few” vs. “many.”

## Introduction

The visual world comes in a bewildering variety of shapes and colors. Since it is impossible to learn the relevant properties of each object one by one, humans and other animals have developed the ability to group stimuli along several dimensions (e.g., Herrnstein and Loveland, 1964; Delius et al., 2000; Makino and Jitsumori, 2007). Usually, members of a category are grouped on the basis of physical similarities. Behaviorally, a category is defined by an ability to generalize within a class of stimuli and to discriminate between classes (Keller and Schoenfeld, 1950), as well as to extrapolate the categorical knowledge to new members of the stimulus class (Wasserman et al., 1988). To date, a large number of demonstrations of successful categorizations in non-human animals have been published. However, in most of these studies performance could simply be based on “categorization by rote” (Vaughan and Greene, 1984; Yamazaki et al., 2007) without requiring an understanding of the abstract relation between the categorized stimuli.

Some methods have been proposed to be critical for proving the establishment of a flexible and abstract relation between stimulus classes (e.g., Astley and Wasserman, 1998). One important technique is the discrimination reversal procedure. It was first proposed by Lea (1984) in order to show concept discriminations, and has since been used in a variety of experiments (e.g., Vaughan, 1988; Von Fersen and Lea, 1990; Delius et al., 1995, 2000), including one which tested a dolphin with auditory stimuli (von Fersen and Delius, 2000). Using a discrimination reversal procedure permits testing whether the subject associates all members of a category even if these members have no common physical property. In a standard reversal procedure, the subject is first trained to discriminate between members from two different categories in a simultaneous discrimination task. After mastering the discrimination, the trained contingencies are reversed in a subset of the employed stimuli. Thus, responses which previously led to reinforcement are now punished, and vice versa. After again reaching discrimination criterion, the new contingency is tested with the remaining members of a group. If the subject spontaneously transposes the reversed contingency to these remaining patterns, it is likely that the animal is able to categorize the members dependent on associations within a category.

Kilian et al. (2003) have previously reported a bottlenose dolphin to be able to discriminate among visual patterns differing in numerosity, i.e., a stimulus property defined by the number of discriminable elements contained in the stimulus. Although it is very likely that dolphins were able to use numerosity to discriminate between different patterns in this experiment, it is not clear if they indeed used a more abstract category based on “few” vs. “many.” Therefore, the present experiment was designed to test for the presence of such an abstract relation when performing a numerical discrimination task. Additionally, we aimed to extend the previously demonstrated range of numbers (1–6) to a larger range (1–10) in order to define the limit of a bottlenose dolphin’s numerical discrimination abilities.

## Materials and Methods

The subject of the present study was an experimentally naive male bottlenose dolphin. At the start of the investigation “Blue” was 10-years old and from birth on almost blind on his right eye. He was housed together with four other bottlenose dolphins in a 13.5 m × 28 m outdoor pool of 4.5 m depth in Marineland Majorca (Spain). The experiments took place in an adjacent pool of 4.45 m × 5.70 m × 1.80 m (*w* × *l* × *d*) in which he was separated from the others during each session.

### General procedure

**Figure 1:**
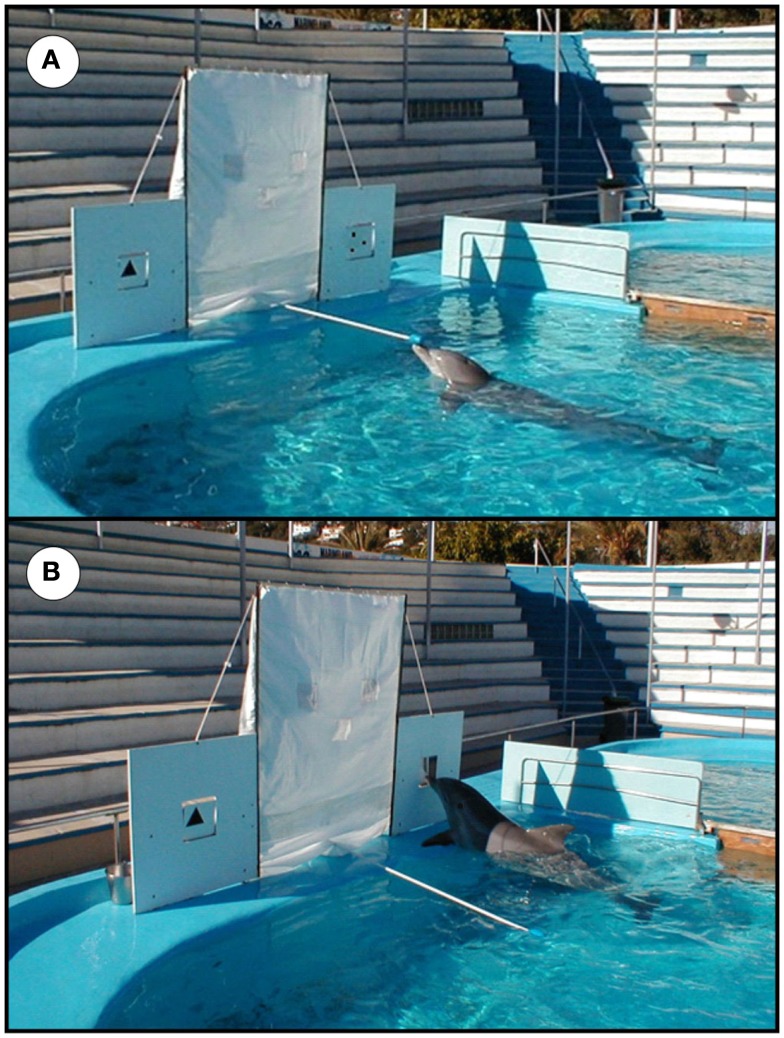
**(A)** Overview of the testing situation with the apparatus, the stationing device, and the position of the dolphin facing the revealed stimuli; **(B)** Blue touching one of the displayed stimuli with his rostrum.

The animal had to discriminate between simultaneously displayed stimuli representing “few” and “many” elements (Figure 1A). The stimuli consisted of 25cm × 25 cm white PVC boards with black items stuck onto them. Each stimulus was inserted in a square-shaped window located on a white painted wooden panel of 1 m^2^. A push with the dolphin’s beak could flip the stimulus backward (Figure 1B). The stimuli were positioned to the left and to the right of the experimenter. The distance between the two panels was 1.50 m. During the discrimination process the experimenter was hidden from the subject’s view by means of a plastic curtain. Each trial started with the animal being positioned at the tip of a 2.50 m target, above water level, and facing the apparatus (Figure 1A). After positioning the animal, the experimenter revealed the covered stimuli and 4 s later indicated by a short whistle that the subject had to leave the target to touch one of the displayed stimuli with its rostrum. Only responses which tipped either stimulus backward were recorded. Correct responses were followed by a continuous whistle blow and reinforced with fish. Incorrect choices were indicated by non-continuous whistle blows and directly followed by correction trials. The position of the correct stimulus (left or right) was alternated quasi-randomly (Gellermann, 1933). The subject was presented with one to two daily sessions of 20 trials each. The only exceptions were the very first presentations of new number pairs, for which a session consisted of 10 trials only to minimize frustration. Criterion was reached after achieving 85% correct performance within a given session.

### Pre-test and habituation phase

Prior to starting the actual experiment, “Blue” received some habituation training with the apparatus. He learned to be sent and wait at the target until the starting signal was given, and then to swim back and touch one of the two white panels. During five sessions of 20 trials each, he was rewarded irrespective of the side the panel he touched was on. This was done to test for a possible side preference. Subsequently, Blue was tested for a possible preference for “few” or “many” items, again in five sessions of 20 trials. To this end, the panels containing few or many items were alternated quasi-randomly, and “Blue” was rewarded after each choice irrespective of which stimulus he had chosen.

## Experiment 1

### Training phase

**Figure 2:**
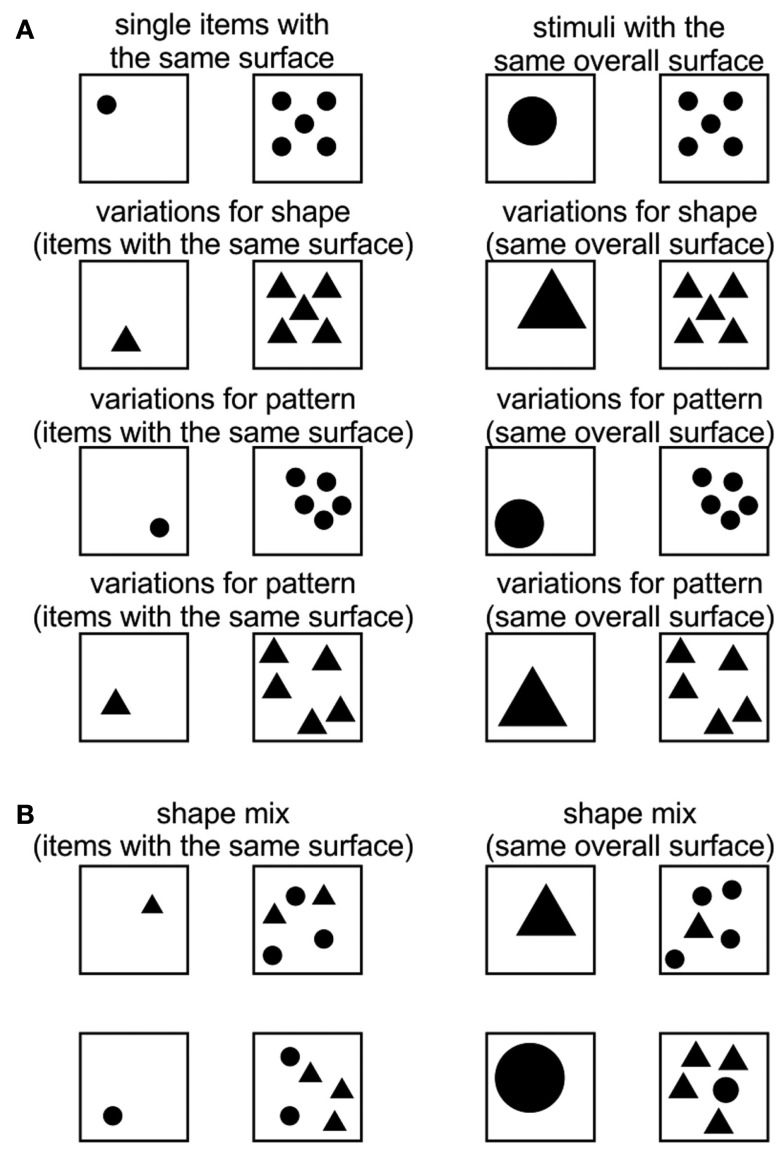
**(A)** Examples of the stimulus pair 1 vs. 5 with variations of surface, size, shape, and pattern of elements. Similar conditions were also used for 1 vs. 4, 1 vs. 3, and 1 vs. 2; **(B)** examples of control stimuli for 1 vs. 5. Similar conditions were presented for 1 vs. 4, 1 vs. 3, and 1 vs. 2.

During training sessions, the animal learned to discriminate 1 vs. 5 and was rewarded for choosing the stimuli which contained more elements. The stimuli consisted of black circles (*r* = 2.4 cm). After reaching 85% correct performance, this stimulus pair was used to habituate “Blue” to unreinforced trials (catch trials). Subsequently, he was trained with the following number pairs: 1 vs. 4, 1 vs. 3, and 1 vs. 2. After successful performance, the animal was also trained with stimulus pairs varying in surface, shape, and element patterns, whereby two different conditions were conducted for the variable “surface”: (1) single items with the same surface, and (2) items having the same overall surface. For “shape,” the initial circles were substituted for triangles. In order to create different “patterns,” the elements were organized in different arrangements (Figure 2A). For each condition (surface, shape, and pattern), five sessions were run, each of which included six catch trials. We did not balance or systematically vary the perimeter of the stimuli, but ensured that in our stimulus set, the overall perimeter was sometimes longer or shorter on the rewarded panel. For example, the perimeters of a single triangle vs. two circles were 27.57 and 30.16 cm, respectively, in one set of panels and 39 and 30.16 cm, respectively, in another.

### Control phase

During control sessions, new stimulus pairs were introduced, mixing the shapes of the elements (circle, triangle, square) for the two panels representing “few” and “many.” Furthermore, variations of up to 100% regarding the size of the elements were introduced (Figure 2B). In a given session, 16 familiar stimulus pairs were mixed with four novel pairs which were never reinforced (catch trials). Moreover, two familiar stimulus pairs were also not reinforced in order to prevent novelty to be exclusively associated with no reward. During this procedure, “Blue” was only confronted with the familiar number combinations of the training phase (1 vs. 5, 1 vs. 4, 1 vs. 3, and 1 vs. 2). He always had to choose the panel containing more elements. These elements could be either circles or triangles or squares, and the total surface of the elements could be the same, smaller, or bigger than for the panel representing “few” elements. In total, 10 sessions were run and 40 new unreinforced stimuli pairs were introduced. Criterion was reached after 85% correct performance had been achieved.

### Testing phase

During the testing phase, new number pairs with new numerosities (2 vs. 5, 3 vs. 5, 2 vs. 4, 2 vs. 3, 3 vs. 4, 4 vs. 5, 5 vs. 6, 5 vs. 7, 5 vs. 9, and 5 vs. 10) were introduced, mixed with training and control stimuli, and tested without feedback (catch trials). As in the control phase, a session consisted of four new number combinations and 16 familiar stimuli of which two were also not reinforced. For each new number pair, five sessions were conducted, and again, variations concerning the shape, size, and pattern were presented. In this phase, we also used outlined and filled elements. In addition, different shapes and sizes were mixed on one panel. Accuracy criterion was again set to 85% correct answers during one session.

### Results of experiment 1

#### Pre-test

Blue showed a clear preference for the left side, choosing left in 70% of trials. When being confronted with panels showing “few” or “many” items that alternated between left and right, he continued to swim left, this time even in 96% of cases. No spontaneous preference for “few” (52%) or “many” elements (48%) could be detected.

#### Training phase

**Figure 3:**
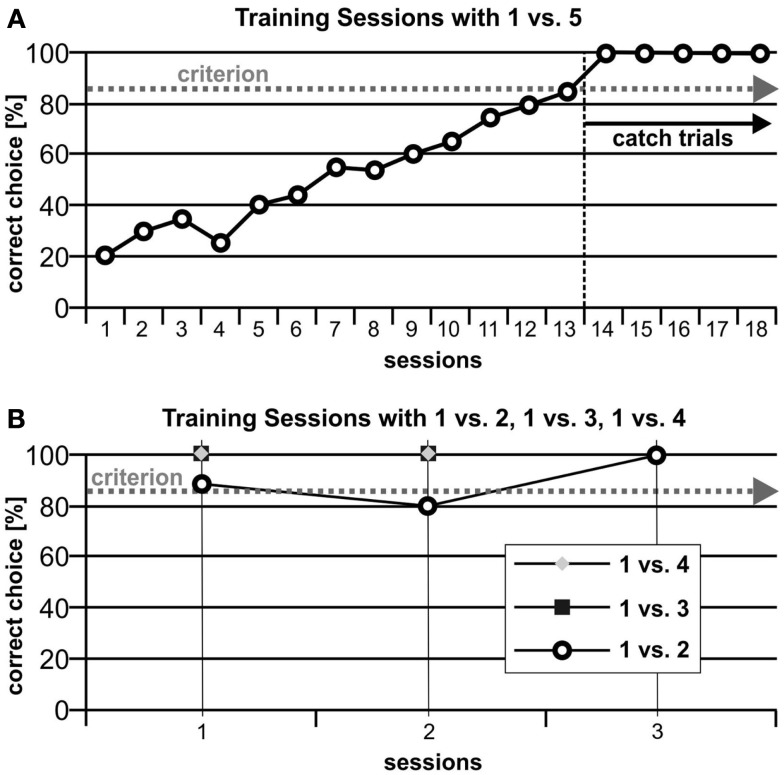
**(A)** For the first stimulus pair 1:5, the subject needed 13 sessions to reach criterion. His performance remained stable even after the introduction of catch trials. **(B)** For the following numerosities, criterion was already reached in the first sessions.

For the first training pair (1 vs. 5), the subject needed 13 sessions to reach criterion. His performance remained stable even after introducing catch trials. For the following training pairs (1 vs. 4, 1 vs. 3, 1 vs. 2), criterion was already reached in the first session. Performance levels remained constant also for pattern, shape, and size variations (Figure 3).

#### Control phase

**Figure 4:**
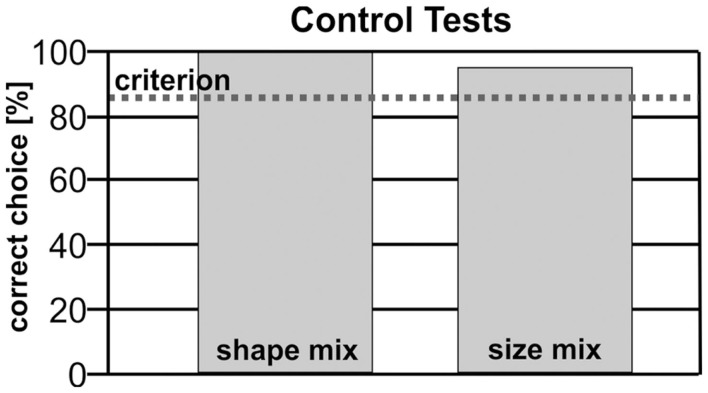
**The results of the control phase show that the animal could transfer the learned performance to new and unreinforced stimuli**.

Blue’s performance for variations of shape, pattern, and surface size was above the criterion level for all conditions (Figure 4).

#### Testing phase

**Figure 5:**
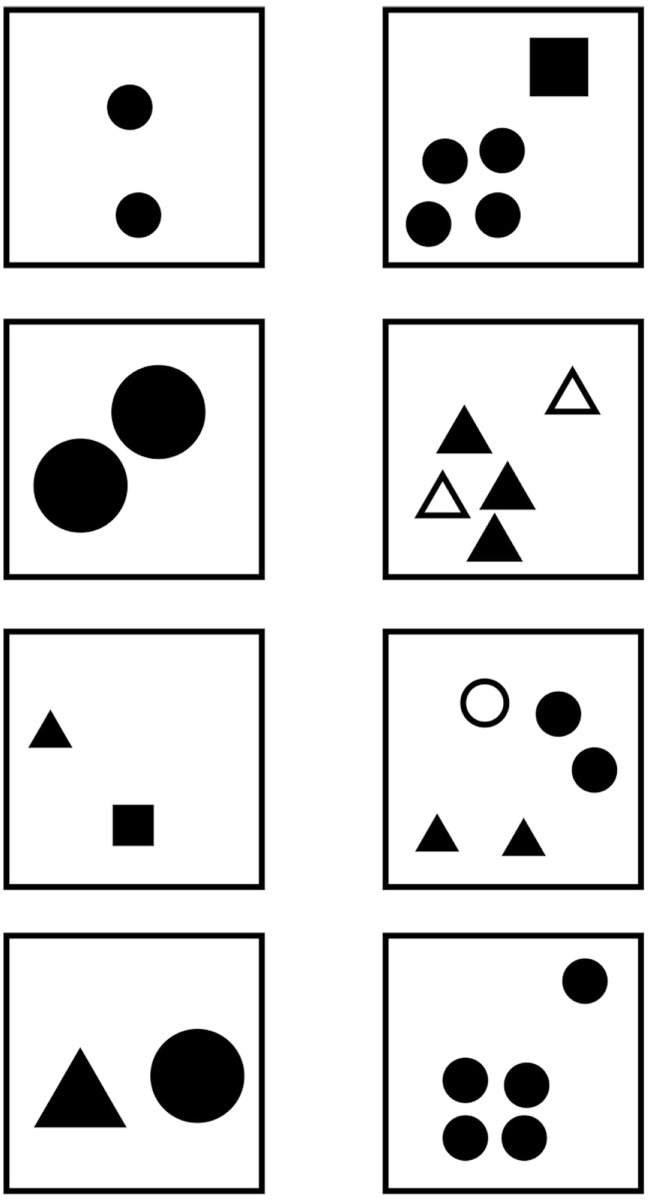
**Examples for the testing stimuli 2 vs. 5**. Similar variations were prepared for the stimuli 3 vs. 5, 3 vs. 5, 2 vs. 4, 2 vs. 3, 3 vs. 4, 4 vs. 5, 5 vs. 6, 5 vs. 7, 5 vs. 9, and 5 vs. 10.

**Figure 6:**
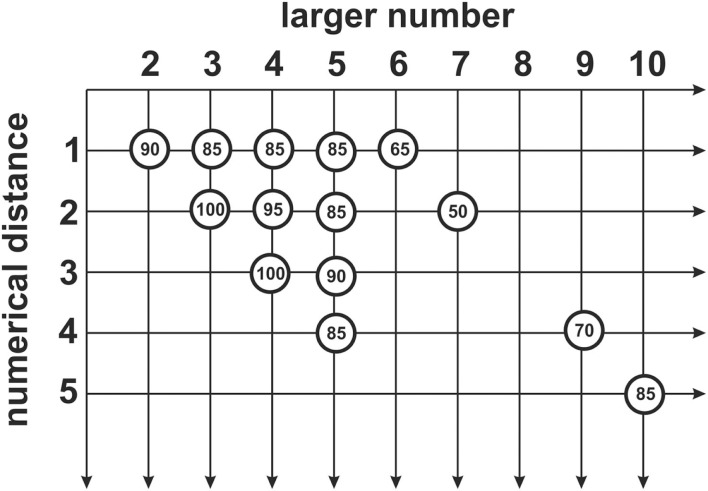
**The figure indicates the larger number within each pair as well as the difference between this pair of stimuli**. The number within the circles indicates the achieved performance of Blue. The data show that Blue had more difficulties in discriminating higher numerosities with small differences between the two numbers.

Blue’s performance for the new and untrained stimuli (Figure 5) 2 vs. 5 was 90%. For 2 vs. 4, he reached 95%, and for 3 vs. 5, 2 vs. 3, 3 vs. 4, and 4 vs. 5 85%. For the combinations 5 vs. 6 (65%), 5 vs. 7 (50%), and 5 vs. 9 (70%), Blue failed to reach criterion. For 5 vs. 10, the criterion was (85%; Figure 6).

### Discussion of experiment 1

The aim of the first experiment was two replicate the results of Kilian et al. (2003), and to test if the numerical range of the previous study (1–6) can be extended to 1–10. Our results clearly replicate Kilian et al. (2003) and demonstrate that numerical competence is in the reach of bottlenose dolphins. Our results are largely in line with a previous study (Mitchell et al., 1985) which showed that a dolphin could choose correctly among the number of fish on a scale from 0 to 5. However, in the study by Mitchell et al. (1985), numerosity was confounded by the amount of food, and the subject could just have perceived the objects as representing hedonic values rather than members of an ordinal series.

At the beginning of the experiments, Blue demonstrated a preference for the left side, possibly due to his right eye being almost blind. A tendency to shift to the sighted side is well known under monocular vision (Ulrich et al., 1999). Blue’s side preference disappeared after being rewarded for selecting the “many” patterns. Overall, Blue’s performance did not appear to be influenced by confounding stimuli like surface, shape, and element patterns. The importance of controlling these factors has been described in several studies on numerical abilities using various species like dolphins (Kilian et al., 2003), pigeons (Emmerton et al., 1997; Xia et al., 2001), monkeys (Cantlon and Brannon, 2007), newborn chicks (Rugani et al., 2011), and human infants (Strauss and Curtis, 1981; Clearfield and Mix, 1999, 2001). These results suggest that, if available, animals including humans may rely on variables that are simpler and therefore less effortful than numerosity (Davis and Memmott, 1982; Beran, 2007). Consequently, Davis and Pérusse (1988) argued that numerosity is the last cognitive resort if other means fail. Along with data from other species (Brannon and Terrace, 1998; Boysen and Hallberg, 2000; Brannon, 2006; Cantlon and Brannon, 2007; Vallortigara et al., 2010), our data clearly argue against this notion, since Blue seemed to spontaneously use numerosity even though other cues were initially available.

This interpretation could also explain why Blue was so rapidly able to generalize to other numerical examples during the control phase without loss of performance (Figure 4). Similar results regarding a transfer to heterogeneous stimulus sets were also found for other animals such as pigeons (Emmerton et al., 1997), a gray parrot (Pepperberg, 1987), a Californian Sea lion (Dieckmann, 1999), rhesus monkeys (Brannon and Terrace, 1998), rats (Suzuki and Kobayashi, 2000), and hooded crows (Smirnova et al., 2000). In the very beginning of the task, Blue could have relied on a strategy to avoid 1. However, the fact that he worked above threshold when being confronted with panels that did not contain the element “1” renders it likely that Blue grasped numerosity as the essence of the task very early on. At least at the present state of analysis of a single subject, our results indicate that for dolphins, numerosity could be a cue that is available before experimental onset (Hauser et al., 2002; Hyde, 2011). In this sense, Blue could reveal a “number sense” (Dehaene et al., 1998).

This last interpretation contrasts with the data of Kilian et al. (2003) who reported Noah, their subject, to completely rely on non-numerical cues in the beginning of the experiment. Although the difference between Blue and Noah could be ascribed to inter-individual differences, other interpretations are also conceivable. Kilian et al. (2003) used three dimensional stimuli consisting of diverse objects in different numbers hanging into water. Noah had to swim from a distance of 10 m and indicate his choice by touching one of the objects. Thus, Noah was confronted with stimuli which provided cues that could be discerned by visual and auditory senses. Additionally, Noah could utilize motion parallax, shape and depth cues, whereas Blue could only use two dimensional vision. It is possible that the comparably more frugal stimulus repertoire of the present study made the spontaneous use of numerosity cues more likely. Thus, dolphins appear to be able to apply a concept of numerosity very early on when encountering stimuli if other cues are less salient. Similar results were obtained by Beran (2007) who tested the influence of non-numerical cues in rhesus monkeys, and by Agrillo et al. (2009) who studied mosquito fish in a 2 vs. 3 object discrimination task, also probing the influence of non-numerical parameters.

Within the limitations of a study conducted with a single animal, the present data suggest that bottlenose dolphins are able to categorize numerosities up to 10. The next experiment was designed as a reversal task in order to test if Blue was able to process a more abstract relation of “few” vs. “many.” According to some authors (Lea, 1984), successful transfer of reversed contingencies to items that were never reversed requires the existence of intra-categorical associations and could even be seen as evidence for a true numerosity concept.

## Experiment 2

### Reversal phase

To evaluate whether Blue indeed had acquired associative bonds between single numerical elements, the animal was confronted with a reversed S+, thus having to decide in favor of the panel with the “few” element. For this purpose, Blue was successively trained with only two numerical combinations: 1 vs. 4 and 1 vs. 5. Blue was already familiar with these numerical combinations from the initial training phase, but this time, reinforcement was delivered after choosing the panel with “1.” After reaching the criterion of 85% correct performance, catch trials with other numerical combinations (1 vs. 3, 2 vs. 3, 3 vs. 4, 3 vs. 6) were intermixed with the two training pairs. The procedure of this phase was the same as described for the test phase. Note that because of poor performance of the number pairing 3 vs. 4 (see Results), the subject received additional training sessions with the training pair 1 vs. 4 and 1 vs. 5 before the combination 3 vs. 6 was tested.

### Results

**Figure 7:**
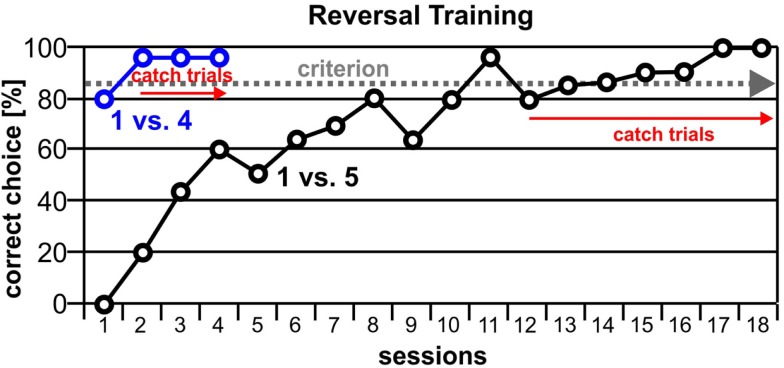
**Blue reached the criterion for the first reversal-training of 1 vs. 5 (85% correct choices) after 11 sessions, and in the following session the introduction of catch trials started**. In the next session, performance dropped to 80% but recovered quickly. For the subsequently introduced training pair 1 vs. 4, criterion was already reached in the second session, and performance remained constant even after the introduction of catch trials.

Blue needed eleven sessions to reach criterion for the first reversal stimuli 1 vs. 5. The performance after the introduction of catch trials initially dropped to 80%, but recovered in the next session and remained constant for the following sessions. Blue reached the criterion for the reversal stimuli 1 vs. 4 already in the second session, and the animal’s performance was constant after the introduction of catch trials (Figure 7).

**Figure 8:**
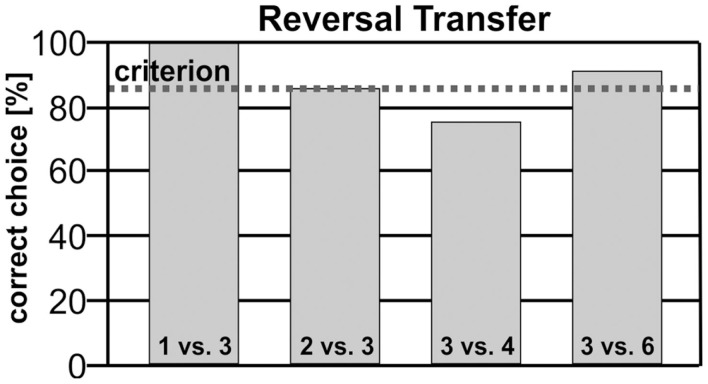
**Blue’s performance for the reversal contingencies 1 vs. 3, 2 vs. 3, 3 vs. 4, and 3 vs. 6**. Only for the stimuli pair 3 vs. 4 did Blue’s performance not reach criterion (75%), albeit his acquisition clearly was above chance level.

Over five sessions, Blue reached 100% correct answers for 1 vs. 3. For the combination 2 vs. 3, he reached 85%, whereby the first four catch trials of the first session were correct. For the stimulus pairing 3 vs. 4, he failed to reach criterion (75% correct performance). For the last number combination 3 vs. 6, Blue reached 90% correct performance (Figure 8).

### Discussion of Experiment 2

The second experiment was designed to test if Blue had already acquired strong intra-categorical associations. As argued by several authors (Lea, 1984; Herrnstein, 1990), members of a category or concept are bound together independent of their perceptual similarities. Thus, contingencies applied to one stimulus of a class should be transferred to all other members. Indeed, Blue was highly successful in this transfer. His performance dropped to 75% only for 3 vs. 4, possibly due to the operations of the analog magnitude system that is subject to a ratio limit in accordance with the Weber-Fechner law (Fechner, 1888; Agrillo et al., 2012). Thus, a simple stimulus generalization can be excluded, since Blue could base his decision only on abstract qualities (few/more). As a further control, we had used new stimuli for the reversal transfer, with a different arrangement of items compared to the first part of the experiment. The possibility that Blue based his decisions on a response outcome is also unlikely, as all new stimuli were introduced by catch trials. Thus, we can also exclude new learning by feedback.

Taken together, the dolphin of the present study demonstrated its capacity to reverse all numerical comparisons after being trained for reversal with only two numerical distinctions. Our results contrast with the results of other authors who reported the necessity of large amounts of training stimuli in non-human animals for developing an abstract concept (Roitblat and von Fersen, 1992; Zentall et al., 2002; Fabre-Thorpe, 2003). Independent of this, we are inclined to conclude that an abstract representation of “few” vs. “many” is within the reach of dolphins.

## General Discussion

In the present work, we examined whether a bottlenose dolphin could rely on a numerical understanding of “few” vs. “many.” Similar to Kilian et al. (2003), we carefully excluded several confounding factors, i.e., that the subject was cued by physical properties of the stimuli other than numerosity. Blue immediately transferred learned contingencies to novel numerical combinations. Data suggest that he likely made the use of a parallel subitizing and an analog magnitude system. Moreover, he was able to reverse the remaining stimulus sets after being exposed to only two number pairings without being taught to do so. Such immediate reversal of performance strongly suggests an abstract understanding of “few” vs. “more” and could even be considered as evidence for a numerosity concept (Lea, 1984). Bottlenose dolphins often aggregate in “super – alliances.” Here, subgroups of males join temporally in order to get numerical advantage over another group to gain access to a receptive female (Hauser, 2000; Connor et al., 2001). Thus, an understanding of magnitude could be of advantage to dolphins living in the wild. In the following, we will discuss the present data in a more general framework.

For magnitudes up to three, Blue could readily discriminate between numerosities that differed by one. Beyond that, his performance started to deteriorate and was just at criterion in experiment 1 or slightly below in experiment 2. This is typical for a “parallel” or subitizing system that only works for small sets up to 3 or 4. Usually, reaction time curves of human subjects that having to judge the number of dots within briefly flashed displays show a monotonic increase with an increase in dot numbers. However, the slopes of these curves display a distinct change at around 3–4 items, for which a fast subitizing process is thought to be succeeded by a true counting mechanism (Trick and Pylyshyn, 1993; Lemer et al., 2003). Below 4, subjects usually accurately discriminate dot numbers despite only brief presentation times and when the ratio of the two numbers is smaller than 1:2.

When being confronted with numerosities beyond 3 or 4, animals seem to process numerical comparisons logarithmically. Indeed, Nieder and Miller (2003) showed that in monkeys, the coding of numerical information follows logarithmic scaling as postulated by the Weber-Fechner law. Thus, with pairings of higher numbers but constant absolute difference, the relative difference becomes smaller and is therefore more difficult to discriminate. Numerous investigations in human infants (Strauss and Curtis, 1981; Xu and Spelke, 2000), human adults (Xu, 2003; Piazza et al., 2004; Hyde and Spelke, 2008; Cordes and Brannon, 2009; Schmitt and Fischer, 2011), human adults with few number words (see citation inside of Brannon, 2006), other primates (Thomas et al., 1980; Boysen, 1993; Boysen and Hallberg, 2000; Smith et al., 2003; Brannon, 2006; Jordan and Brannon, 2006; van Marle et al., 2006; Addessi et al., 2007; Beran, 2007; Cantlon and Brannon, 2007; Hanus and Call, 2007; Nieder and Merten, 2007; Beran et al., 2008), pigeons (Scarf et al., 2012), New Zealand robins (Hunt et al., 2008), and domestic chicks (Rugani et al., 2008) show similar results. Agrillo et al. (2012) observed this distinction in comparable ways in undergraduate students and guppies, and argued for the existence of two numerical systems that have a long phylogenetic history. However, the existence of two systems is not undisputed. Some authors present evidence that most experimental data can be explained by a single magnitude system (Nieder, 2005; Nieder and Merten, 2007). Alternatively, subitizing could mainly occur in studies in which subjects use behavioral discriminations by accessing implicit representations of the number of objects (Hauser et al., 2000).

We set out to study if numerosity in dolphins is represented as a flexible and abstract category representing the more or the less of a magnitude. To this end, we employed the partial reversal procedure in which only a subset of numerosities is reversed and the remainders are subsequently tested. According to Lea (1984) and Herrnstein (1990), successful partial reversal can signal the presence of a numerosity concept. Indeed, Blue successfully switched his choices after single reversal learning. Thus, within the limits a study conducted with only a single individual, we are inclined to believe that bottlenose dolphins can flexible represent numerosity as an abstract magnitude system. This result is similar to another dolphin study in which two dolphins were shown to categorize “same” vs. “different” for different visual objects (Mercado et al., 2000). Numerical competence at a level similar to Blue has previously also been shown for monkeys and parrots (Matsuzawa, 1985; Pepperberg, 1987). A successful mastery of abstract category use in monkeys was described by Bovet and Vauclair (2001). In this study, animals had to judge two objects as same or different and afterward transfer their learned skills to new objects which belonged to two functional categories (food/non-food). Other examples are provided by flexible token use in capuchin monkeys as described by Addessi et al. (2007), or by achievement of abstract relations like “inside-outside” (Herrnstein et al., 1989). The parallel results of cognitive capacities of dolphins and primates, other mammals and birds despite their different evolutionary history and ecology reveal that vertebrates uses the same basic and evolutionary old processes when flexibly dealing with categories (Mercado et al., 2000). Results like these argue in favor of a continuous evolutionary process of cognitive competences, an evolutionary process for which humans represent an integral part of the overall pattern (Vauclair, 2002; Pepperberg and Gordon, 2005; Diester and Nieder, 2007).

## Conflict of Interest Statement

The authors declare that the research was conducted in the absence of any commercial or financial relationships that could be construed as a potential conflict of interest.
